# An approach for clustering gene expression data with error information

**DOI:** 10.1186/1471-2105-7-17

**Published:** 2006-01-12

**Authors:** Brian Tjaden

**Affiliations:** 1 Computer Science Department, Wellesley College, Wellesley, MA 02481, USA

## Abstract

**Background:**

Clustering of gene expression patterns is a well-studied technique for elucidating trends across large numbers of transcripts and for identifying likely co-regulated genes. Even the best clustering methods, however, are unlikely to provide meaningful results if too much of the data is unreliable. With the maturation of microarray technology, a wealth of research on statistical analysis of gene expression data has encouraged researchers to consider error and uncertainty in their microarray experiments, so that experiments are being performed increasingly with repeat spots per gene per chip and with repeat experiments. One of the challenges is to incorporate the measurement error information into downstream analyses of gene expression data, such as traditional clustering techniques.

**Results:**

In this study, a clustering approach is presented which incorporates both gene expression values and error information about the expression measurements. Using repeat expression measurements, the error of each gene expression measurement in each experiment condition is estimated, and this measurement error information is incorporated directly into the clustering algorithm. The algorithm, CORE (Clustering Of Repeat Expression data), is presented and its performance is validated using statistical measures. By using error information about gene expression measurements, the clustering approach is less sensitive to noise in the underlying data and it is able to achieve more accurate clusterings. Results are described for both synthetic expression data as well as real gene expression data from *Escherichia coli *and *Saccharomyces cerevisiae*.

**Conclusion:**

The additional information provided by replicate gene expression measurements is a valuable asset in effective clustering. Gene expression profiles with high errors, as determined from repeat measurements, may be unreliable and may associate with different clusters, whereas gene expression profiles with low errors can be clustered with higher specificity. Results indicate that including error information from repeat gene expression measurements can lead to significant improvements in clustering accuracy.

## Background

The maturation of microarray technology in recent years has provided researchers with large amounts of gene expression data requiring computational analysis. One approach which has proven useful in elucidating trends in this data is clustering, an algorithmic technique for partitioning genes into groups which evince similar expression patterns. Since most formulations of clustering problems are NP-hard, clustering algorithms tend to focus on approximation methods. For example, hierarchical clustering [1], *k*-means [2], graph-theoretic approaches [3], and self-organizing maps [4] are examples of heuristically motivated clustering methods which have been applied to gene expression data. As an alternative, model-based clustering methods assume that the expression data can be modelled by a set of distributions [5,6], most commonly as a finite mixture of multivariate Gaussian distributions [7-10]. The abovementioned clustering methods are generally unsupervised, and they can be distinguished from supervised classification of gene expression data, which occurs when clusters or groups are *known *for some subset of gene expression data, and the known examples can be used as training data. Examples of supervised classification techniques include multilayer perceptrons [11] and support vector machines [12]. Model-based approaches have also been applied in the context of supervised classification [13]. In this study, we focus on the problem of unsupervised clustering, as true classes for gene expression patterns are rarely known *a priori*.

The clustering approach proposed in this study is most closely related to the *k*-means algorithm. In the statistics and machine learning literature, *k*-means is one of the most popular clustering methods, in part because of its efficiency, and consequently, a wealth of research has investigated extensions to the algorithm. For example, semi-supervised versions of *k*-means which incorporate background knowledge into the algorithm have been studied [14,15]. Methods for choosing the initial seeds or starting points for the algorithm have also proven successful [16,17]. While *k*-means can be formulated as minimizing a sum-of-squares function, reformulations have been investigated which allow more effective searches for function minima [18,19]. Also, variations of pairwise distance measures, which are employed by the algorithm, have been studied [20].

In the process of partitioning genes into groups, most clustering approaches, either explicitly or implicitly, calculate pairwise distances or similarities between pairs of gene expression profiles. For researchers interested in applying clustering techniques to gene expression data, often the choice of pairwise distance (or similarity) measure is as important as the choice of clustering approach. The two most common measures for gene expression data are Euclidean distance and Pearson correlation [21]. While correlation is a similarity measure rather than a distance measure, it can be converted to a dissimilarity measure (i.e., distance measure) through a straightforward transformation. The Euclidean distance between two gene expression profiles reflects the magnitude of difference between the profiles, whereas the correlation of two profiles reflects the similarity in shape, or pattern, between the profiles. Consequently, the correlation measure is invariant to linear transformations in gene expression patterns. Gibbons and Roth compared these two measures and found that correlation performed best on non-ratio based gene expression data, whereas Euclidean distance performed best on ratio-based gene expression data [21]. These researchers suggested that Euclidean distance outperforms correlation on ratio-based data because ratio-based data are generally log-transformed, thereby equalizing up and down regulation effects and compressing the scale of variation. In the algorithm proposed in this study, we employ a measure which, in the general case, accounts for linear transformations in the data, analogous to correlation. The proposed algorithm can also cluster based on Euclidean distance, as a special case of the general distance measure.

One of the main challenges for all of the clustering techniques, regardless of the pairwise distance measure, is the substantial noise in the underlying data sets [22,23]. Expression measurements from microarray experiments can range dramatically in their accuracy and reproducibility [24-26]. Even the best clustering methods are unlikely to provide meaningful results if too much of the data is unreliable. In recent years, there has been a wealth of work done on statistical analysis of gene expression data, including selecting sets of relevant features or genes [27-29], modelling errors and uncertainty in array measurements [30-33], and investigating the effects of repeat experiments on accuracy and reproducibility of expression data [34,35], to name a few. With increasing emphasis on modelling error information in gene expression data, researchers are designing more experiments with repeat gene expression measurements. One of the challenges, then, is to incorporate measurement error information into downstream analyses of gene expression data, such as clustering.

An area related to the use of error information in clustering is the use of error information in identifying differentially expressed genes. Given the expression levels for a set of genes in two different tissues or at two different time points, we may ask which genes in the set are differentially expressed under the two conditions. Several studies have incorporated error information, as derived from replicated gene expression measurements, into methods for identifying differentially expressed genes [36-40]. In general, these approaches conduct a hypothesis test for each gene, and then correct for multiple tests. Often, these methods are variations of a *t*-test, and they differ primarily in their estimation of the variance.

We are concerned with a somewhat different problem in this study, namely how error information, as determined from repeat gene expression measurements, can improve traditional clustering techniques. Previously, the incorporation of error information into clustering algorithms has been investigated in the context of model-based clustering. Medvedovic and Sivaganesan [41] proposed a mixture model for clustering gene expression data with error information. While, initially, the mixture model assumed that expression measurement errors for each gene were homogeneous across experiments, the model has been extended to include different error estimates across the experiments [10,42]. One of the advantages of the mixture model is the generality of the model, which enables its applicability for a range of data sets [42]. However, the model generality comes at the price of having many parameters necessitating estimation. For instance, to estimate a covariance matrix for each of *k *mixture components (i.e., clusters), k*m^2 ^parameters must be determined, where *m *is the number of experiment conditions. In addition, the model does not currently account for linear transformations of gene expression profiles. In other words, the model considers the magnitude of gene expression profiles (analogous to Euclidean distance) but not the *pattern *(or shape) of gene expression profiles (analogous to correlation). In contrast, the approach proposed in this study, CORE, is a heuristic algorithm which performs clustering based on the pattern of gene expression profiles.

Rather than evolve a clustering algorithm to incorporate error information, as is proposed in this study, an alternative approach for including error information is to modify the input to standard clustering methods. Since many clustering algorithms take as input a matrix of pairwise similarities (or distances) between all pairs of gene expression profiles, the pairwise similarity matrix may be modified to include error information. This approach has the advantage of utilizing traditional clustering algorithms, such as hierarchical clustering, *k*-means, and self-organizing maps, without modification.

In an early study, Hughes *et al*. [43] estimate the errors of gene expression measurements for their yeast compendium data set using, in part, replicate assays. The authors then incorporate these error estimates as they calculate a weighted similarity (analogous to a correlation coefficient) of each gene's expression pattern with every other gene's expression pattern. The correlation calculation is weighted based on the estimated errors of the gene expression measurements. The result is an *n × n *similarity matrix, where *n *is the number of genes to be clustered. Each entry in the matrix corresponds to an error-weighted similarity measure between two genes. The matrix is then input into a hierarchical clustering algorithm. Thus, the authors do not alter their clustering algorithm to include the error information, but rather they modify their pairwise similarity measure. Other approaches [42,44], more recently, have employed error-weighted similarity measures based on replicate expression assays in their clustering applications. Again, traditional clustering algorithms are used, but the input to the algorithms – an *n *× *n *similarity matrix for *n *gene expression profiles – is calculated using an error-weighted similarity measure as opposed to traditional Euclidean distances or correlation coefficients.

The error-weighted similarity is useful because it represents a single measure for each pair of genes, based on both error information and gene expression values, which can be input into existing clustering methods. However, as we demonstrate in our results, the approach is problematic in two respects. Firstly, because expression measurements and the resulting error estimates are reduced to a pairwise similarity matrix before being input to a clustering algorithm, some error information is lost. In particular, the approach captures the *relative *error between different experiment measurements when calculating similarity of genes, but it does not capture the *absolute *measurement errors between genes. In other words, it captures experiment specific noise but not gene specific noise. Gene specific errors may be the result of various biases in microarray assays, such as sequences hybridizing with different affinities or mRNAs exhibiting different levels of stability or rates of degradation. Secondly, and more importantly, the error-weighted similarity measure is not a true measure of gene expression correlation, i.e., it does not necessarily capture similarity between gene expression profiles. Rather, it represents the similarity between ratios of expression level to estimated error. Generally, gene expression profiles (i.e., genes with similar expression patterns) are the desired targets of clustering applications, not ratios of expression to error, which have no clear biological interpretation.

We present the clustering algorithm, CORE (Clustering of Repeat Expression data), a clustering approach akin to *k*-means clustering with measurement error information included intrinsically. Using repeat expression measurements, a single surrogate expression value is calculated for each gene in each experiment condition, and the error is estimated for each of these surrogate gene expression values. This error information is then incorporated into the clustering model, enabling CORE to capture much of the noise in the underlying data sets, both from experiment biases and gene biases. A higher weight is placed on reliable expression measurements and less weight is placed on unreliable expression measurements when clustering. By identifying and down-weighting noisy measurements, more accurate clusterings are achieved. The performance of CORE is validated using statistical measures, and clustering results are presented for synthetic expression data sets as well as real gene expression data from *Escherichia coli *and *Saccharomyces cerevisiae*. All data as well as supplementary information is available at the website, .

## Results and discussion

### Error models

**Figure 1:**
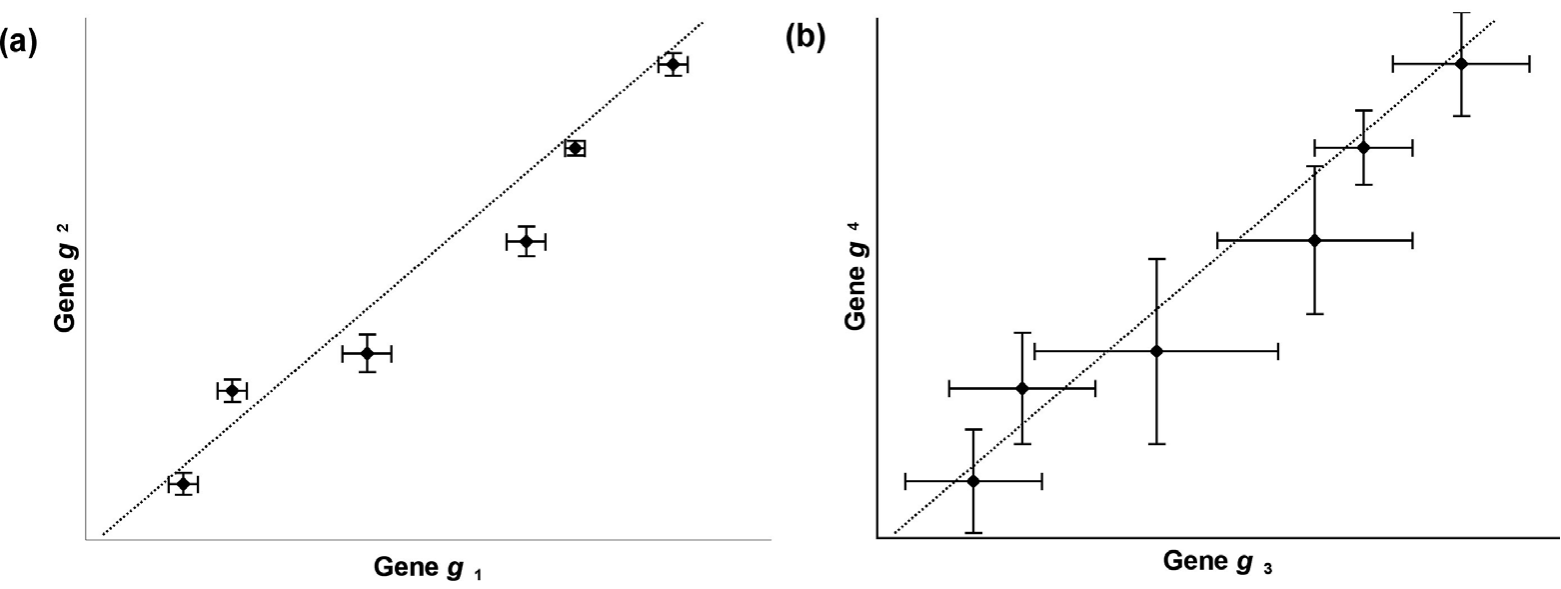
**Scatter plots of gene expression profiles. **(a) A scatter plot of the expression profiles for 2 genes (with 6 components) with standard errors indicated, (b) A scatter plot of the expression profiles for 2 genes (with 6 components) identical to the expression profiles in (a), but with higher standard errors. The gene pairs in (a) and (b) have identical Euclidean distances, identical correlation coefficients, and identical error-weighted similarity. However, in the CORE clustering algorithm, genes whose expression measurements have higher error (*g*_3 _or *g*_4_) provide less information about which cluster the gene belongs to, and the gene makes less of a contribution toward the calculation of clustering parameters.

As microarray assays increasingly are performed with replicate experiments or replicate spots per gene on each array, our ability to capture standard error information associated with each measured expression value in each condition is also improving [31,45,46]. Incorporating this error information can improve the effectiveness of clustering analysis [47]. For instance, consider the expression profiles for four genes (g_1_, *g*_2_, *g*_3_, and *g*_4_) where the expression measurements for two of the genes (*g*_1 _and *g*_2_) have very low error and the expression measurements for the other two genes (*g*_3 _and *g*_4_) have higher absolute error, as in Fig. (1). If *g*_1 _and *g*_3 _have identical expression profiles and *g2 *and *g4 *have identical expression profiles then most clustering algorithms will calculate the same distance or similarity (assuming Euclidean distance or correlation similarity) for g_1 _and *g*_2 _as for g_3 _and *g*_4 _because most clustering algorithms do not incorporate error information about expression measurements. Even in the case of the previously discussed error-weighted similarity measure [42-44], the similarity between the two pairs of genes in Fig. (1) is identical because the gene pairs have the same relative errors across experiments.

In general, the previously proposed error-weighted similarity measure is problematic because it is not a true measure of correlation between gene expression patterns. An error-weighted similarity measure for two genes *x *and *y *across *m *experiments (i.e., components) has been described as

**Figure 2:**
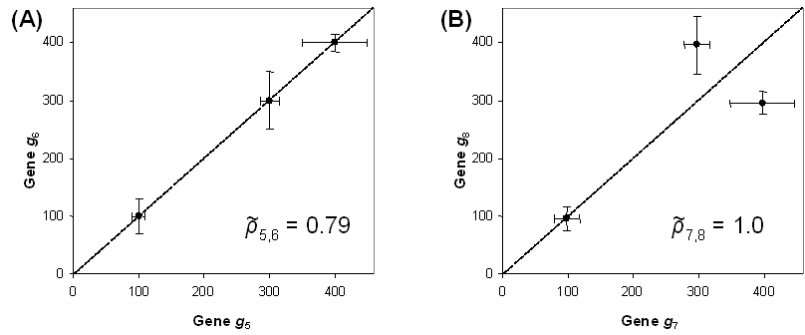
**Error-weighted similarity examples. **The figures (A) and (B) depict examples when error-weighted similarity (Eq. (1) in the text) is problematic as a correlation measure. (A) A scatter plot of the expression profiles for two genes g5 and g6 (with 3 components), g5 = (100, 300, 400) and g6 = (100, 300, 400). The plotted expression profiles fall exactly on a straight line, however the error-weighted similarity ρ˜5,6
 MathType@MTEF@5@5@+=feaafiart1ev1aaatCvAUfKttLearuWrP9MDH5MBPbIqV92AaeXatLxBI9gBaebbnrfifHhDYfgasaacH8akY=wiFfYdH8Gipec8Eeeu0xXdbba9frFj0=OqFfea0dXdd9vqai=hGuQ8kuc9pgc9s8qqaq=dirpe0xb9q8qiLsFr0=vr0=vr0dc8meaabaqaciaacaGaaeqabaqabeGadaaakeaaiiGacuWFbpGCgaacamaaBaaaleaacqaI1aqncqGGSaalcqaI2aGnaeqaaaaa@3180@ for these genes is only 0.79 when 05 = (10, 15, 50) and *σ *6 = (30, 50, 15). (B)A scatter plot of the expression profiles for two genes g7 and g8 (with 3 components), g7 = (100, 300, 400) and g8 = (100, 400, 300). The plotted expression profiles do not fall on a straight line, however the error-weighted similarity ρ˜7,8
 MathType@MTEF@5@5@+=feaafiart1ev1aaatCvAUfKttLearuWrP9MDH5MBPbIqV92AaeXatLxBI9gBaebbnrfifHhDYfgasaacH8akY=wiFfYdH8Gipec8Eeeu0xXdbba9frFj0=OqFfea0dXdd9vqai=hGuQ8kuc9pgc9s8qqaq=dirpe0xb9q8qiLsFr0=vr0=vr0dc8meaabaqaciaacaGaaeqabaqabeGadaaakeaaiiGacuWFbpGCgaacamaaBaaaleaacqaI3aWncqGGSaalcqaI4aaoaeqaaaaa@3188@ for these genes is 1.0 when 01 = (20, 20, 50) and *σ *8 = (20, 50, 20).

ρ˜xy=∑j=1m(gxj−μxσxj)(gyj−μyσyj)(∑j=1mgxj−μxσxj)2(∑j=1mgyj−μyσyj)2, where μi=∑j=1mgjiσij∑j=1m1σij     (1)
 MathType@MTEF@5@5@+=feaafiart1ev1aaatCvAUfKttLearuWrP9MDH5MBPbIqV92AaeXatLxBI9gBaebbnrfifHhDYfgasaacH8akY=wiFfYdH8Gipec8Eeeu0xXdbba9frFj0=OqFfea0dXdd9vqai=hGuQ8kuc9pgc9s8qqaq=dirpe0xb9q8qiLsFr0=vr0=vr0dc8meaabaqaciaacaGaaeqabaqabeGadaaakeaaiiGacuWFbpGCgaacamaaBaaaleaacqWG4baEcqWG5bqEaeqaaOGaeyypa0ZaaSaaaeaadaaeWbqaamaabmaabaWaaSaaaeaacqWGNbWzdaWgaaWcbaGaemiEaGNaemOAaOgabeaakiabgkHiTiab=X7aTnaaBaaaleaacqWG4baEaeqaaaGcbaGae83Wdm3aaSbaaSqaaiabdIha4jabdQgaQbqabaaaaaGccaGLOaGaayzkaaWaaeWaaeaadaWcaaqaaiabdEgaNnaaBaaaleaacqWG5bqEcqWGQbGAaeqaaOGaeyOeI0Iae8hVd02aaSbaaSqaaiabdMha5bqabaaakeaacqWFdpWCdaWgaaWcbaGaemyEaKNaemOAaOgabeaaaaaakiaawIcacaGLPaaaaSqaaiabdQgaQjabg2da9iabigdaXaqaaiabd2gaTbqdcqGHris5aaGcbaWaaOaaaeaadaqadaqaamaaqahabaWaaSaaaeaacqWGNbWzdaWgaaWcbaGaemiEaGNaemOAaOgabeaakiabgkHiTiab=X7aTnaaBaaaleaacqWG4baEaeqaaaGcbaGae83Wdm3aaSbaaSqaaiabdIha4jabdQgaQbqabaaaaaqaaiabdQgaQjabg2da9iabigdaXaqaaiabd2gaTbqdcqGHris5aaGccaGLOaGaayzkaaWaaWbaaSqabeaacqaIYaGmaaGcdaqadaqaamaaqahabaWaaSaaaeaacqWGNbWzdaWgaaWcbaGaemyEaKNaemOAaOgabeaakiabgkHiTiab=X7aTnaaBaaaleaacqWG5bqEaeqaaaGcbaGae83Wdm3aaSbaaSqaaiabdMha5jabdQgaQbqabaaaaaqaaiabdQgaQjabg2da9iabigdaXaqaaiabd2gaTbqdcqGHris5aaGccaGLOaGaayzkaaWaaWbaaSqabeaacqaIYaGmaaaabeaaaaGccqGGSaalcqqGGaaicqqG3bWDcqqGObaAcqqGLbqzcqqGYbGCcqqGLbqzcqqGGaaicqWF8oqBdaWgaaWcbaGaemyAaKgabeaakiabg2da9maalaaabaWaaabCaeaadaWcaaqaaiabdEgaNnaaBaaaleaacqWGQbGAcqWGPbqAaeqaaaGcbaGae83Wdm3aaSbaaSqaaiabdMgaPjabdQgaQbqabaaaaaqaaiabdQgaQjabg2da9iabigdaXaqaaiabd2gaTbqdcqGHris5aaGcbaWaaabCaeaadaWcaaqaaiabigdaXaqaaiab=n8aZnaaBaaaleaacqWGPbqAcqWGQbGAaeqaaaaaaeaacqWGQbGAcqGH9aqpcqaIXaqmaeaacqWGTbqBa0GaeyyeIuoaaaGccaWLjaGaaCzcamaabmaabaacbeGae4xmaedacaGLOaGaayzkaaaaaa@B43B@

where *g*_*ij *_is the estimated expression of gene *i *in experiment *j *and *σ*_*ij *_is its associated error [42-44]. As Fig. (2) illustrates, this similarity measure can be problematic because it does not necessarily capture similarity between gene expression patterns, i.e., correlation. Using the error-weighted similarity measure in Eq. (1), two genes' expression values may fall along a straight line through the origin (be perfectly correlated), but they may have an error-weighted correlation of less than 1.0 (the error-weighted correlation is 0.79 for the gene pair in Fig. (2a)). Alternatively, two genes' expression values may not fall along a straight line (may not be perfectly correlated), yet they may have an error-weighted correlation of 1.0, as in Fig. (2b). This problem is due to the fact that the weighted correlation formula does not compute the correlation of a two-dimensional data distribution (which would have to assign a single weight to each (*x, y*) pair). Instead of computing a weighted correlation between expression levels, it computes a correlation between ratios of expression level to standard error, leading to a correlation number that does not necessarily represent similarity of expression pattern, as illustrated in Fig. (2).

In order to avoid these pitfalls, we incorporate error information into our clustering model intrinsically. Intuitively, if a gene's expression measurements have very high error then the gene expression profile provides little information about which cluster it belongs to, and the gene should make very little contribution to clustering calculations. Inversely, if a gene's expression measurements have very low error then we may have greater confidence in the gene's expression profile, and the gene should make a greater contribution to the clustering. As an example, in the limit as the error approaches infinity, a gene's expression measurement is equally likely to take any value, i.e., it is equally likely to belong to any cluster. In order to account for this variability in measurements, the assignment of a gene to a cluster is determined as a function not only of the distance between the gene and cluster but also of the standard errors of the gene's expression measurements. Further, different experiments may result in different levels of measurement error *for a given gene*. Since the proposed approach considers the error for each gene in each experiment, noisy coordinates of a gene's expression profile can be identified and the clustering contribution of these coordinates can be downweighted accordingly. This approach reduces the weight of noisy expression measurements so that CORE is less sensitive to the uncertainty and error which is common in array experiments.

### Clustering model

Suppose we have gene expression values for *n *genes over *m *conditions, and each one of these *n × m *values has an associated error, calculated from replicate assays. In the most straightforward strategy, each gene expression value may be determined as the mean over repeat measurements, and the associated error may be the sample standard error over the repeat measurements. A number of alternative strategies exist for estimating expression values and errors which can capture various statistical properties of the experiments [31,32,45]. The proposed clustering algorithm is an extension of the *k*-means heuristic. As a frame of reference, the *k*-means algorithm is a special case of the finite mixture model where the *k *underlying probability distributions are assumed to be Gaussians, all with equal variance and uniform prior probabilities [48,49]. In the proposed approach, each cluster is modelled by a set of parameters describing an expression profile over *m *coordinates for the cluster. The algorithm for clustering iterates between two alternating steps. In the first step, model parameters are estimated for each cluster conditioned on the set of genes assigned to the cluster. In the second step, cluster assignments for each gene are determined conditioned on the clusters' model parameters. In each step, the parameter estimations are a function of both gene expression values and their corresponding measurement errors.

### Linear transformation

**Figure 3:**
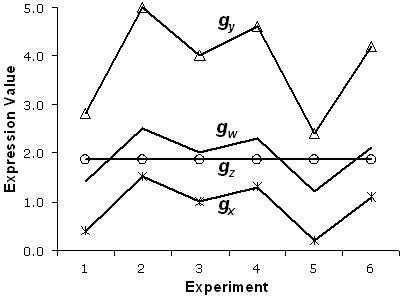
**Transformed gene expression profiles. **Four gene expression profiles across six experiments are depicted. The CORE algorithm uses two parameters, *β*_*i *_and *γ*_*i*_, for each gene to reflect linear transformations of a gene's expression profile. The parameter *β *represents multiplicative scaling and the parameter *γ *represents additive translation. In the figure, the expression profile for *g*_*x *_is a translated version (*β *= 1, *γ *= -1) of the profile for *g*_*w*_, and the profile for *g*_*y *_is a scaled version (*β *= 2, *γ *= 0) of that for *g*_*w*_. Thus, the three expression profiles, *g*_*w*_, *g*_*x *_and *g*_*y*_, have the same shape, and all three are perfectly correlated, i.e., have a distance of zero from each other in the CORE algorithm. In contrast, the profiles for *g*_*w *_and *g*_*z *_have different shapes but are the closest in terms of Euclidean distance.

The model assumes that each gene's observed expression pattern is generated from one of the *k *expression profiles, with a linear transformation that involves multiplicative scaling and/or additive translation of an observation. In common practice, the expression profiles for two genes are considered to be the same (i.e., have a pairwise distance of zero) if the profiles are linear transformations of each other. The Pearson correlation coefficient is often used as a measure of pairwise similarity between two expression profiles in traditional clustering approaches precisely because it accounts for linear transformations between profiles (i.e., two expression profiles which are linear transformations of each other are perfectly correlated). Alternatively, if Euclidean distance is used as a pairwise distance metric, appropriate normalization of Euclidean pairwise distances will account for linear transformations (i.e., the distance will be zero between two expression profiles which are linear transformations of each other). However, as described above, error information is lost when correlation (or normalized Euclidean distance) is calculated *ab initio*. Thus, the proposed algorithm does not calculate pairwise similarities (or distances) between expression profiles. In order to retain error information about each gene in each experiment, linear transformations are accounted for *within *the clustering algorithm so that error information is not lost. For each gene, two parameters, *β *and *γ*, are used which explicitly model multiplicative scaling and additive translation respectively. To illustrate the idea of linear transformations, Fig. (3) shows the expression profiles for four genes, *g*_*w*_, *g*_*x*_, *g*_*y *_and *g*_*z*_, across six experiments. The expression profiles for *g*_*w *_and *g*_*z *_have different shapes, but they are the closest pair in terms of Euclidean distance. In contrast, the expression profile for *g*_*x *_is a translated version (*β *= 1, *γ *= -1) of the profile for *g*_*w*_, and the profile for *g*_*y *_is a scaled version (*β *= 2, *γ *= 0) of that for *g*_*w*_. Hence, the three expression profiles, *g*_*w*_, *g*_*x *_and *g*_*y*_, have the same shape, and all three are perfectly correlated. For applications in which standard (non-normalized) Euclidean distances are preferred, the linear transformation parameters can be set appropriately (*β *= 1, *γ *= 0).

### Clustering formalism

Let *g*_*ij *_be the expression value for gene *i *in experiment *j*, and let *σ*_*ij *_be the standard error corresponding to this expression value as determined from repeat measurements. Then the expression profile of a gene *i*, generated from a cluster with profile *α *= (*α*_1_, ..., *α*_*m*_), is described as

*g*_*ij *_= *β*_*i*_*α*_*j *_+ *γ*_*i *_+ *ε*_*ij *_    (2)

where *ε*_*ij *_are independent error terms with mean 0 and variance σji2
 MathType@MTEF@5@5@+=feaafiart1ev1aaatCvAUfKttLearuWrP9MDH5MBPbIqV92AaeXatLxBI9gBaebbnrfifHhDYfgasaacH8akY=wiFfYdH8Gipec8Eeeu0xXdbba9frFj0=OqFfea0dXdd9vqai=hGuQ8kuc9pgc9s8qqaq=dirpe0xb9q8qiLsFr0=vr0=vr0dc8meaabaqaciaacaGaaeqabaqabeGadaaakeaaiiGacqWFdpWCdaqhaaWcbaGaemOAaOMaemyAaKgabaGaeGOmaidaaaaa@324D@ Here, *β*_*i *_is the scaling factor and *γ *_*i *_is the translation factor of gene *i*. The variability may be stabilized by dividing by *σ*_*ij *_to obtain

gijσij=βiαjσij+γiσij+εijσij.     (3)
 MathType@MTEF@5@5@+=feaafiart1ev1aaatCvAUfKttLearuWrP9MDH5MBPbIqV92AaeXatLxBI9gBaebbnrfifHhDYfgasaacH8akY=wiFfYdH8Gipec8Eeeu0xXdbba9frFj0=OqFfea0dXdd9vqai=hGuQ8kuc9pgc9s8qqaq=dirpe0xb9q8qiLsFr0=vr0=vr0dc8meaabaqaciaacaGaaeqabaqabeGadaaakeaadaWcaaqaaiabdEgaNnaaBaaaleaacqWGPbqAcqWGQbGAaeqaaaGcbaacciGae83Wdm3aaSbaaSqaaiabdMgaPjabdQgaQbqabaaaaOGaeyypa0Jae8NSdi2aaSbaaSqaaiabdMgaPbqabaGcdaWcaaqaaiab=f7aHnaaBaaaleaacqWGQbGAaeqaaaGcbaGae83Wdm3aaSbaaSqaaiabdMgaPjabdQgaQbqabaaaaOGaey4kaSYaaSaaaeaacqWFZoWzdaWgaaWcbaGaemyAaKgabeaaaOqaaiab=n8aZnaaBaaaleaacqWGPbqAcqWGQbGAaeqaaaaakiabgUcaRmaalaaabaGae8xTdu2aaSbaaSqaaiabdMgaPjabdQgaQbqabaaakeaacqWFdpWCdaWgaaWcbaGaemyAaKMaemOAaOgabeaaaaGccqGGUaGlcaWLjaGaaCzcamaabmaabaWexLMBbXgBcf2CPn2qVrwzqf2zLnharyGvLjhzH5wyaGabbiaa+ndaaiaawIcacaGLPaaaaaa@63C4@

Hence the error terms

gij−(βiαj+γi)σij     (4)
 MathType@MTEF@5@5@+=feaafiart1ev1aaatCvAUfKttLearuWrP9MDH5MBPbIqV92AaeXatLxBI9gBaebbnrfifHhDYfgasaacH8akY=wiFfYdH8Gipec8Eeeu0xXdbba9frFj0=OqFfea0dXdd9vqai=hGuQ8kuc9pgc9s8qqaq=dirpe0xb9q8qiLsFr0=vr0=vr0dc8meaabaqaciaacaGaaeqabaqabeGadaaakeaadaWcaaqaaiabdEgaNnaaBaaaleaacqWGPbqAcqWGQbGAaeqaaOGaeyOeI0IaeiikaGccciGae8NSdi2aaSbaaSqaaiabdMgaPbqabaGccqWFXoqydaWgaaWcbaGaemOAaOgabeaakiabgUcaRiab=n7aNnaaBaaaleaacqWGPbqAaeqaaOGaeiykaKcabaGae83Wdm3aaSbaaSqaaiabdMgaPjabdQgaQbqabaaaaOGaaCzcaiaaxMaadaqadaqaamXvP5wqSXMqHnxAJn0BKvguHDwzZbqegyvzYrwyUfgaiqqacaGF0aaacaGLOaGaayzkaaaaaa@50D4@

have mean 0 and variance 1. Thus, the distance from gene *i *to a profile *α *is given by

min⁡βi,γi∑j=1m[gij−(βiαj+γi)σij]2     (5)
 MathType@MTEF@5@5@+=feaafiart1ev1aaatCvAUfKttLearuWrP9MDH5MBPbIqV92AaeXatLxBI9gBaebbnrfifHhDYfgasaacH8akY=wiFfYdH8Gipec8Eeeu0xXdbba9frFj0=OqFfea0dXdd9vqai=hGuQ8kuc9pgc9s8qqaq=dirpe0xb9q8qiLsFr0=vr0=vr0dc8meaabaqaciaacaGaaeqabaqabeGadaaakeaadaWfqaqaaiGbc2gaTjabcMgaPjabc6gaUbWcbaacciGae8NSdi2aaSbaaWqaaiabdMgaPbqabaWccqGGSaalcqWFZoWzdaWgaaadbaGaemyAaKgabeaaaSqabaGcdaaeWbqaamaadmaabaWaaSaaaeaacqWGNbWzdaWgaaWcbaGaemyAaKMaemOAaOgabeaakiabgkHiTiabcIcaOiab=j7aInaaBaaaleaacqWGPbqAaeqaaOGae8xSde2aaSbaaSqaaiabdQgaQbqabaGccqGHRaWkcqWFZoWzdaWgaaWcbaGaemyAaKgabeaakiabcMcaPaqaaiab=n8aZnaaBaaaleaacqWGPbqAcqWGQbGAaeqaaaaaaOGaay5waiaaw2faaaWcbaGaemOAaOMaeyypa0JaeGymaedabaGaemyBa0ganiabggHiLdGcdaahaaWcbeqaaiabikdaYaaakiaaxMaacaWLjaWaaeWaaeaatCvAUfeBSjuyZL2yd9gzLbvyNv2CaeHbwvMCKfMBHbaceeGaa4xnaaGaayjkaiaawMcaaaaa@669B@

which is equivalent to maximum likelihood estimation for independent *ε*_*ij *_assuming that they are normally distributed, a reasonable assumption based on previous studies [7,45]. Similarly, given a set of genes assigned to *α *cluster *C*, the cluster profile *a *for *C *can be determined by

arg⁡ min(α1,α2,…,αm){∑i∈Cmin⁡βi,γi∑j=1m[gij−(βiαj+γi)σij]2}.     (6)
 MathType@MTEF@5@5@+=feaafiart1ev1aaatCvAUfKttLearuWrP9MDH5MBPbIqV92AaeXatLxBI9gBaebbnrfifHhDYfgasaacH8akY=wiFfYdH8Gipec8Eeeu0xXdbba9frFj0=OqFfea0dXdd9vqai=hGuQ8kuc9pgc9s8qqaq=dirpe0xb9q8qiLsFr0=vr0=vr0dc8meaabaqaciaacaGaaeqabaqabeGadaaakeaadaWfqaqaaiGbcggaHjabckhaYjabcEgaNjabbccaGiabb2gaTjabbMgaPjabb6gaUbWcbaGaeiikaGccciGae8xSde2aaSbaaWqaaiabigdaXaqabaWccqWFSaalcqWFXoqydaWgaaadbaGaeGOmaidabeaaliab=XcaSiablAciljab=XcaSiab=f7aHnaaBaaameaacqWGTbqBaeqaaSGaeiykaKcabeaakmaacmaabaWaaabuaeaadaWfqaqaaiGbc2gaTjabcMgaPjabc6gaUbWcbaGae8NSdi2aaSbaaWqaaiabdMgaPbqabaWccqGGSaalcqWFZoWzdaWgaaadbaGaemyAaKgabeaaaSqabaaabaGaemyAaKMaeyicI4Saem4qameabeqdcqGHris5aOWaaabCaeaadaWadaqaamaalaaabaGaem4zaC2aaSbaaSqaaiabdMgaPjabdQgaQbqabaGccqGHsislcqGGOaakcqWFYoGydaWgaaWcbaGaemyAaKgabeaakiab=f7aHnaaBaaaleaacqWGQbGAaeqaaOGaey4kaSIae83SdC2aaSbaaSqaaiabdMgaPbqabaGccqGGPaqkaeaacqWFdpWCdaWgaaWcbaGaemyAaKMaemOAaOgabeaaaaaakiaawUfacaGLDbaaaSqaaiabdQgaQjabg2da9iabigdaXaqaaiabd2gaTbqdcqGHris5aOWaaWbaaSqabeaacqaIYaGmaaaakiaawUhacaGL9baacqGGUaGlcaWLjaGaaCzcamaabmaabaWexLMBbXgBcf2CPn2qVrwzqf2zLnharyGvLjhzH5wyaGabbiaa+zdaaiaawIcacaGLPaaaaaa@8712@

The objective of the algorithm is to determine *k *clusters such that the sum of distances from each gene to its closest cluster profile is minimized. If *δ*_*i *_is the distance of gene *i *to its closest cluster profile, then the objective function Δ is given by

Δ=∑i=1nδi.     (7)
 MathType@MTEF@5@5@+=feaafiart1ev1aaatCvAUfKttLearuWrP9MDH5MBPbIqV92AaeXatLxBI9gBaebbnrfifHhDYfgasaacH8akY=wiFfYdH8Gipec8Eeeu0xXdbba9frFj0=OqFfea0dXdd9vqai=hGuQ8kuc9pgc9s8qqaq=dirpe0xb9q8qiLsFr0=vr0=vr0dc8meaabaqaciaacaGaaeqabaqabeGadaaakeaacqGHuoarcqGH9aqpdaaeWbqaaGGaciab=r7aKnaaBaaaleaacqWGPbqAaeqaaaqaaiabdMgaPjabg2da9iabigdaXaqaaiabd6gaUbqdcqGHris5aOGaeiOla4IaaCzcaiaaxMaadaqadaqaamXvP5wqSXMqHnxAJn0BKvguHDwzZbqegyvzYrwyUfgaiqqacaGF3aaacaGLOaGaayzkaaaaaa@4837@

**Figure 4:**
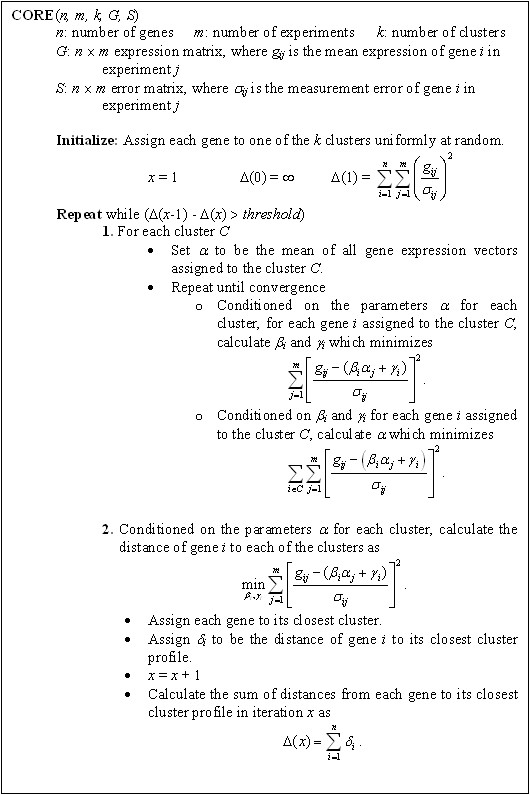
**CORE algorithm. **The figure provides a description of the CORE algorithm.

The algorithm is a gradient descent procedure which iterates two steps until a locally optimal clustering is achieved [50]. First, parameters are estimated for each cluster (Exp. (6)), and then each gene is assigned to its closest cluster (minimizing Exp. (5) over all clusters). In the former step, the model parameters of each cluster and the transformation parameters for each gene are simultaneously estimated. A description of the algorithm is given in Fig. (4). The runtime of each iteration is linear in the size of the input (i.e., proportional to *n *× *m*), and for large data sets (10,000 genes assayed over 100 experiments), the algorithm runs in a matter of seconds on standard desktop computers.

### Measure of performance

Following the convention of Yeung *et al*. [7], we use the term *cluster *to mean a partition of genes predicted by the algorithm. We use the term *class *to denote a partition of genes which are known to group together by some external evaluation criteria. Under this framework, the accuracy of a particular clustering can be validated against external evaluation criteria, using a metric such as the Rand index [51], *R*. The Rand index also serves as a measure for the consistency of two different clusterings of the same data set. While the expected value of the Rand index for two random clusterings does not take a constant value, the *adjusted *Rand index [52], *R*_*a*_, provides a metric for comparing two clustering results which is designed to correct for the presence of chance agreement in clusterings. Assuming the generalized hypergeometric distribution as the model of randomness, the expected value of *R*_*a *_for two random clusterings is 0. The adjusted Rand index is used to assess the performance of the proposed clustering approach. Earlier studies which compared various statistical measures to validate clustering results [7,53,54] suggest that the adjusted Rand index *R*_*a *_is one of the best measures of cluster validation.

### Validating clustering results

To test the effectiveness of the approach, we implemented the algorithm and ran it on both synthetic and real data sets. For all data sets, the clustering algorithm was applied both with the CORE error model, where errors are estimated for each gene expression value in each experiment, as well as with a uniform error model. The uniform error model represents the case when all expression measurements are assumed to have the same error, as in the case of traditional clustering approaches. The actual value of the measurement error in the uniform error model is unimportant (except for its effect on the constant cluster, as described in Methods) as long as the value is positive and constant, since it is effectively normalized across all genes in all experiments. In addition to the uniform error model, two other alternatives to the CORE model were considered. First, the pairwise Euclidean distance, rather than the linearly transformed pairwise distance, was employed. To calculate the Euclidean distance between expression profiles, the CORE algorithm sets the linear transformation parameters, *β *and *γ*, to fixed values of 1 and 0, respectively, rather than estimating these two parameters. Second, the error-weighted similarity measure was used. The error-weighted similarity measure between expression profiles is calculated by the CORE algorithm using Equation (1). For both synthetic and real data sets, all four variations (CORE error, uniform error, Euclidean distance, error-weighted similarity) were compared.

**Figure 5:**
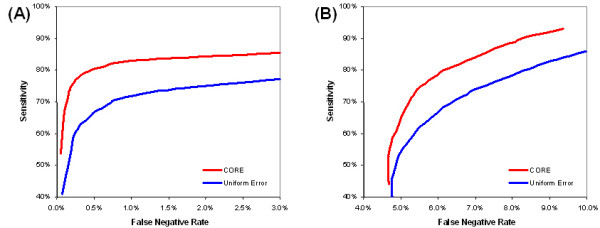
**ROC curve for synthetic data. **The ROC (receiver operating characteristic) curves show the tradeoffs between sensitivity and specificity (i.e., 1.0 - false negative rate) as the number of clusters is varied with two synthetic data sets. At each point along the curve, the sensitivity and specificity values are calculated as an average over 100 trials of generating synthetic data with a given number of classes and clustering the data with the same number of clusters as classes. (A) For the normally distributed synthetic expression data, the number of clusters is varied between 20 and 200. (B) For the periodic time series synthetic expression data, the number of clusters is varied between 2 and 20. The top curve (CORE) uses estimated standard error information from repeat measurements whereas the bottom curve uses a uniform error model, as described in the text.

**Figure 6:**
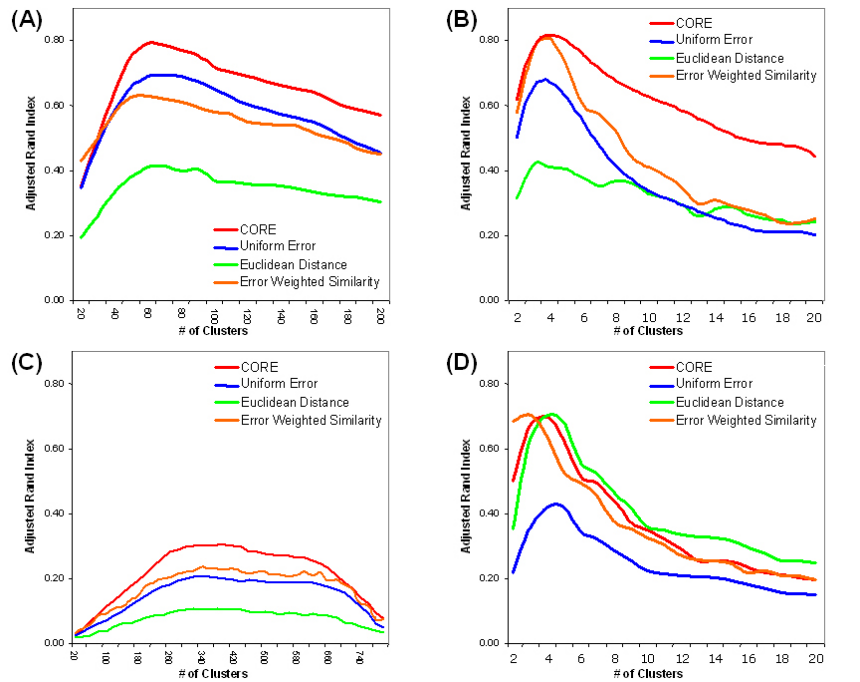
**Adjusted Rand index for synthetic data and real expression data. **Each curve reflects the average adjusted Rand index *R*_*a *_of clustering quality as the number of clusters is varied. Each data point on a curve is an average over 100 trials of generating and clustering data. Four clustering variations are considered for each data set: the CORE error model, a uniform error model, a Euclidean distance between pairs of expression profiles, and the error-weighted similarity measure between pairs of expression profiles. (A) The figure depicts the results for normally distributed synthetic data generated from 50 classes. (B) The figure depicts the results from periodic time series synthetic data generated from 4 classes. (C) The figure shows the results of clustering 904 *E. coli *genes belonging to 275 multi-gene operons based on expression data from 55 experiments. (D) Based on expression data from 20 experimental conditions, the figure shows the results of clustering 205 yeast genes which have each been annotated with one of four functional classifications.

The first synthetic data set corresponds to normally distributed repeat measurements. The number of genes, the number of experiments, the number of replicates, and the errors are all chosen to approximate the *E. coli *data set described below. Synthetic expression data are generated from normal distributions (as described in Methods) for *n *= 1000 genes over *m *= 50 experiments with *ω *= 14 degrees of freedom (corresponding to 15 measurements per gene in each experiment as in the *E. coli *gene expression data sets) and scaling parameter *τ *= 0.1 (as defined in Exp. (9)). For a given number of clusters, 100 trials of generating and clustering synthetic data are conducted, and the average over the 100 trials is calculated. Each synthetic data set is clustered both with the CORE error model as well as with a uniform error model. As the receiver operating characteristic (ROC) curve shows in Fig. (5a), the CORE error model consistently outperforms the uniform error model as the number of clusters is varied from 20 to 200. Here, the number of clusters equals the number of classes at each point on the curve. The ordinate in Figure 5 represents the sensitivity as the number of clusters is varied and the abscissa represents the false negative rate (i.e., 1.0 - specificity). Similarly, in Fig. (6a), synthetic data is generated (as described in Methods) for *n *= 1000 genes over *m *= 50 experiments with *ω *= 14 degrees of freedom and scaling parameter *τ *= 0.1 (Exp. (9)). Here, the number of classes is fixed at 50 and the number of clusters is varied up to 200. In addition to the uniform error model, the CORE model is evaluated using a Euclidean distance measure and using an error-weighted similarity measure. For this data set, the CORE model outperforms the other three. The poor performance of the Euclidean distance measure, in this example, is unsurprising since each synthetic expression profile is generated as a linear transformation of a cluster profile *a*, and the Euclidean distance does not account for linear transformations between profiles.

The second synthetic data set corresponds to periodic time-series measurements. The number of genes, the number of experiments, the number of replicates, and the errors are all chosen to approximate the *S. cerevisiae *(yeast) data set described below. Synthetic expression data are generated from sine waves (as described in Methods) for *n *= 200 genes over *m *= 20 experiments with *ω *= 3 degrees of freedom (corresponding to 4 measurements per gene in each experiment as in the yeast gene expression data sets) and scaling parameter *τ *= 0.1 (as defined in Exp. (9)). For a given number of clusters, 100 trials of generating and clustering synthetic data are conducted, and the average over the 100 trials is calculated. Each synthetic data set is clustered both with the CORE error model as well as with a uniform error model. The ROC curve in Fig. (5b) shows that the CORE error model outperforms the uniform error model as the number of clusters is varied from 2 to 20. Here, the number of clusters equals the number of classes at each point on the curve. Similarly, in Fig. (6b), synthetic data is generated (as described in Methods) for *n *= 200 genes over m = 20 experiments with *ω *= 3 degrees of freedom and scaling parameter *τ *= 0.1 (Exp. (9)). The number of classes is fixed at 4 and the number of clusters is varied up to 20. Again, the CORE model outperforms the others. The poor performance of the Euclidean distance measure is explained by the fact that synthetic expression profiles were generated as linear transformations of cluster profiles, and the Euclidean distance does not account for such linear transformations.

For a set of *E. coli *gene expression data, the clustering approach was validated using 904 genes which have been experimentally verified as belonging to multi-gene operons [55,56]. The expression of each gene in each experiment was measured by 15 probes. These genes belong to 275 operons and serve as a reasonable external standard under the assumption that genes belonging to the same operon should be co-regulated and should belong to the same class. Admittedly, this standard is imperfect. Different operons may be co-regulated leading to an exaggerated number of false positive classifications. Additionally, genes belonging to the same operon may be co-transcribed and co-regulated under some conditions and individually regulated and transcribed under other conditions, thereby inflating the number of false negatives. Nonetheless, we find polycistronic mRNAs to be an excellent overall indication of genes with similar expression patterns, i.e., genes which should cluster together. Fig. (6c) illustrates the results of clustering the set of *E. coli *genes, varying the number of clusters up to 800. For this data set, the CORE model has the best performance.

As a final validation, the clustering approach was applied to a set of yeast expression data [57]. The data consists of expression measurements for 205 genes involved in galactose utilization (GAL) in *Saccharomyces cerevisiae*. Gene expression was measured with 4 replicate assays across 20 experimental conditions (20 perturbations in the GAL pathway). Each of the 205 genes has been annotated as corresponding to one of four functional classifications in the gene ontology [58]. These functional classifications serve as an external standard of classification. It is worth noting that this data set has been clustered previously [10,42]. Fig. (6d) illustrates the results of clustering the set of yeast genes using the CORE algorithm, varying the number of clusters up to 20. For this data set, the Euclidean distance measure outperforms the CORE model. Indeed, these results are consistent with those obtained in a previous study [21], namely that for particular gene expression data sets, Euclidean distance serves as a better measure, when clustering, than correlation or other measures which capture linear transformations in the data. The data sets for which the Euclidean distance may be the most appropriate measure are ratio-based gene expression data, such as the abovementioned yeast expression data (obtained from two-channel cDNA microarray experiments). In contrast, for non-ratio based gene expression data, such as the abovementioned *E. coli *expression data (obtained from single channel microarray experiments), linear transformation measures have been reported to outperform Euclidean distance measures in clustering applications [21]. In support of these findings, Fig. (6c), which is based on non-ratio style data from Affymetrix oligonucleotide array experiments, shows that the linear transformation measure outperforms the Euclidean distance measure for the single channel data set.

In all cases above, the performance of the CORE error model dominates that of the uniform error model and the error-weighed similarity measure. These results are consistent with previous work which compared the effectiveness of the error-weighted similarity measure in clustering [42]. Further investigation of these results confirmed our expectations about the model, namely that genes whose expression measurements have high error could not be assigned to clusters reliably. For these high-error genes, the distribution of distances from the gene to each cluster is relatively flat, i.e., a high error gene has nearly uniform probability of being generated from each cluster. As a result, the noisy measurements contribute less to the clustering. Since the approach considers the error for each gene in each experiment, noisy coordinates could be identified in a given gene's expression profile (i.e., for each gene, experiments which result in higher expression measurement error). As detailed in the algorithm, the less reliable experiments contributed less to the clustering calculations. As shown in the results, this additional error information leads to improvements in clustering accuracy.

### Choosing the number of clusters

One of the challenges with many clustering applications is determining the correct number of clusters (i.e., the choice for the parameter *k*). The problem of choosing an appropriate number of clusters for a given data set has been reviewed by Milligan and Cooper [59] and by Gordon [60]. Several heuristics have been developed for various clustering approaches, such as the gap statistic [61] which is commonly used for heuristic clustering methods and the BIC (Bayesian Information Criterion) [62] which is commonly used for model-based methods [63,64]. The gap statistic, which compares the change in within-cluster dispersion with that expected under an appropriate reference null distribution, has been used previously with *k*-means type algorithms on gene expression data sets [61], and consequently, we demonstrate that it is a useful guide to selecting an appropriate number of clusters in the context of the CORE algorithm presented in this study.

**Figure 7:**
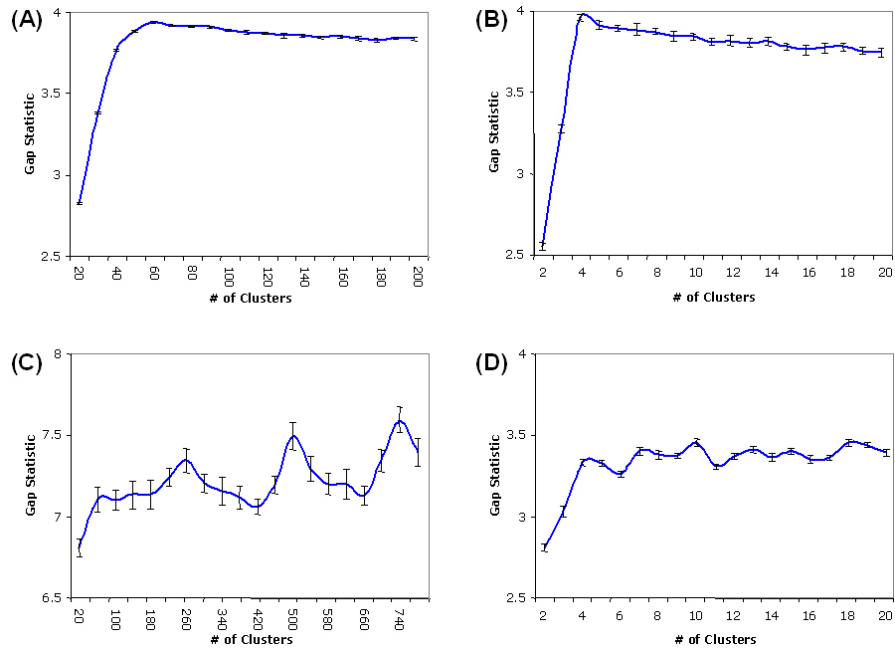
**Gap statistic for estimating the number of clusters. **The figure shows the results of calculating the gap statistic for different numbers of clusters using the CORE algorithm for each of the four data sets. Each point along the curves represents a comparison between the within-cluster dispersion of the clustered data set, as determined by the CORE algorithm, and the average within-cluster dispersion of *B *= 100 samples of a clustered complete reference distribution, as determined by CORE. Generation of the reference distribution is described in the text. The error bars in the figure reflect the standard deviation of the gap statistic across the *B *reference distribution samples. The recommended value for the parameter *k *is the smallest number of clusters, *i*, such that the gap statistic at *i *is greater than or equal to the gap statistic at *i*+1, less the estimated error of the gap statistic at *i*+1. (A) For normally distributed synthetic data generated from 50 classes, the gap statistic suggests a parameter value of *k *= 60. (B) For periodic time series synthetic data generated from 4 classes, the gap statistic suggests a parameter value of *k *= 4. (C) For 904 *E. coli *genes belonging to 275 multi-gene operons, the gap statistic suggests a parameter value of *k *= 260. (D) For 205 yeast genes which have each been annotated with one of four functional classifications, the gap statistic suggests a parameter value of *k *= 5. For each of the four data sets, the gap statistic suggests a value for the parameter *k *which is close to the number of true classes in the data set.

Fig. (7) shows the results of calculating the gap statistic for different numbers of clusters using the CORE algorithm for each of the four data sets. The gap statistic depends on an appropriate reference null distribution, which is generated here as described in previous work [61]. In summary, for each experimental condition, each simulated gene expression measurement from the reference distribution is generated uniformly over the range of observed values for that experimental condition. Each point along the curves in Fig. (7) represents a comparison between the within-cluster dispersion of the clustered data set, as determined by the CORE algorithm, and the average within-cluster dispersion of *B = *100 samples of the clustered complete reference distribution, as determined by CORE. The error bars in the figure reflect the standard deviation of the gap statistic across the *B *reference distribution samples. It is important to note that the recommended value for the parameter *k *is *not *the point at which the gap statistic achieves its global maximum. Rather, it is the smallest number of clusters at which the gap statistic achieves a *local *maximum, after accounting for the error terms. Specifically, the recommended value for *k *is the smallest number of clusters, *i*, such that the gap statistic at *i *is greater than or equal to the gap statistic at *i*+1, less the estimated error of the gap statistic at *i*+1. The non-monotone behaviour of the gap statistic often indicates smaller sub-clusters within larger well-separated clusters. For each of the four data sets, the gap statistic in Fig. (7) suggests a value for the parameter *k *which is close to the number of true classes in the data set.

## Conclusion

As microarray technology matures, arrays are becoming cheaper and denser. In addition, a wealth of research on statistical analysis of gene expression data encourages researchers to consider error and uncertainty in their microarray experiments, so that experiments are being performed increasingly with repeat spots per gene per chip and with repeat experiments. The additional information provided by replicate gene expression measurements is a valuable asset in effective clustering. Gene expression profiles with high standard errors, as determined from repeat measurements, may be unreliable and may fit with many clusters, whereas gene expression profiles with low standard errors can be clustered with higher specificity. A novel clustering approach (CORE) is presented which incorporates measurement error information for gene expression data. The performance of CORE is validated using statistical measures on both synthetic and real gene expression data sets. The results indicate that the inclusion of error information can lead to significant improvements in clustering accuracy as well as decreased sensitivity to noise in the underlying data. All results, as well as the expression data sets are available as supplemental material.

## Methods

### Non-differentially expressed genes

Throughout a set of gene expression experiments, a substantial number of genes may display nearly constant expression patterns across all conditions. Often this is the result of the genes' lack of differential expression under the assayed conditions. In particular, at very low expression levels, the ratio of measurement error to expression value is relatively high. However, since most clustering approaches use expression *patterns *(direction and shape as opposed to magnitude) to cluster, these non-differentially expressed genes with proportionally high error can heavily bias clustering results. To account for these genes, a preprocessing phase is performed of removing any genes demonstrating constant expression across the assayed experiments. These genes are identified by calculating, for each gene *i*, the distance of its expression profile from a constant expression pattern

min⁡γi∑j=1m(gij−γiσij)2.     (8)
 MathType@MTEF@5@5@+=feaafiart1ev1aaatCvAUfKttLearuWrP9MDH5MBPbIqV92AaeXatLxBI9gBaebbnrfifHhDYfgasaacH8akY=wiFfYdH8Gipec8Eeeu0xXdbba9frFj0=OqFfea0dXdd9vqai=hGuQ8kuc9pgc9s8qqaq=dirpe0xb9q8qiLsFr0=vr0=vr0dc8meaabaqaciaacaGaaeqabaqabeGadaaakeaadaWfqaqaaiGbc2gaTjabcMgaPjabc6gaUbWcbaacciGae83SdC2aaSbaaWqaaiabdMgaPbqabaaaleqaaOWaaabCaeaadaqadaqaamaalaaabaGaem4zaC2aaSbaaSqaaiabdMgaPjabdQgaQbqabaGccqGHsislcqWFZoWzdaWgaaWcbaGaemyAaKgabeaaaOqaaiab=n8aZnaaBaaaleaacqWGPbqAcqWGQbGAaeqaaaaaaOGaayjkaiaawMcaaaWcbaGaemOAaOMaeyypa0JaeGymaedabaGaemyBa0ganiabggHiLdGcdaahaaWcbeqaaiabikdaYaaakiabc6caUiaaxMaacaWLjaWaaeWaaeaatCvAUfeBSjuyZL2yd9gzLbvyNv2CaeHbwvMCKfMBHbaceeGaa4hoaaGaayjkaiaawMcaaaaa@5A1C@

Thus, a distribution is obtained, for the *n *genes, of the distance of the expression profiles from a constant expression pattern. By comparing this distribution to a Chi-squared distribution with *m*-ldegrees of freedom, any genes whose expression patterns are sufficiently close to constant (below some threshold) can be identified and removed prior to clustering.

### Data sets

Two types of data are used to assess the performance of the clustering approach. Synthetic data is useful because the *classes *from which each gene expression profile is generated are known exactly, and thus, the results can easily be assessed. Real expression data sets are more relevant, yet it is often difficult to assess these clustering results because there are limited external evaluation criteria for true *classes *of genes.

### Synthetic expression data

Two methods are employed for generating synthetic expression data which capture much of the variability and error of real gene expression data. In the first method, an expression profile *α *for each of *k *classes is initially generated. The expression profiles are vectors chosen uniformly at random from the unit hypercube in *m *dimensional space. For each of *n *genes, one of the *k *classes is randomly chosen, and using the expression profile *a *for the chosen class, the expression value *g*_*ij *_for gene *i *in experiment *j *is determined as *g*_*ij *_= *β*_*i*_*α*_*j *_+ *γ*_*i *_+ *ε*_*ij *_where *β*_*i *_and *γ*_*i *_are chosen uniformly at random. The error *ε*_*ij *_is randomly selected from *N*(0, σji2
 MathType@MTEF@5@5@+=feaafiart1ev1aaatCvAUfKttLearuWrP9MDH5MBPbIqV92AaeXatLxBI9gBaebbnrfifHhDYfgasaacH8akY=wiFfYdH8Gipec8Eeeu0xXdbba9frFj0=OqFfea0dXdd9vqai=hGuQ8kuc9pgc9s8qqaq=dirpe0xb9q8qiLsFr0=vr0=vr0dc8meaabaqaciaacaGaaeqabaqabeGadaaakeaaiiGacqWFdpWCdaqhaaWcbaGaemOAaOMaemyAaKgabaGaeGOmaidaaaaa@324D@). The standard error values *σ*_*ij *_for each component *j *of *g*_*i *_are chosen from the following Chi-squared distribution,

τ1ωχω2     (9)
 MathType@MTEF@5@5@+=feaafiart1ev1aaatCvAUfKttLearuWrP9MDH5MBPbIqV92AaeXatLxBI9gBaebbnrfifHhDYfgasaacH8akY=wiFfYdH8Gipec8Eeeu0xXdbba9frFj0=OqFfea0dXdd9vqai=hGuQ8kuc9pgc9s8qqaq=dirpe0xb9q8qiLsFr0=vr0=vr0dc8meaabaqaciaacaGaaeqabaqabeGadaaakeaaiiGacqWFepaDdaGcaaqaamaalaaabaGaeGymaedabaGae8xYdChaaiab=D8aJnaaDaaaleaacqWFjpWDaeaacqaIYaGmaaaabeaakiaaxMaacaWLjaWaaeWaaeaatCvAUfeBSjuyZL2yd9gzLbvyNv2CaeHbwvMCKfMBHbaceeGaa4xoaaGaayjkaiaawMcaaaaa@4405@

where *ω *indicates the degrees of freedom (corresponding to the number of repeat expression measurements per gene) and *τ *is a scaling parameter. The Chi-squared distribution is an appropriate choice for independent error terms which are normally distributed.

In the second method, an expression profile *a *for each of the *k *classes is generated such that the expression profiles emulate periodic time series data. For the z^th ^class, 1 ≤ *z *≤ *k*, an expression profile *α*(*z*) corresponding to a sine wave for the class is generated. Each class's expression profile (i.e., sampled sine wave) is determined uniquely according to the following function *α*_*j*_(*z*) = sin(2n*(*z*/*k *+ 3*j*/*m*)). The *z/k *term determines the unique shift (along the abscissa) of each sine function. Since *j *represents one of the *m *experiments, 1 ≤ *j *≤ *m*, the term 3*j*/*m *indicates that the experimental coordinates of the expression profile are sampled uniformly from the sine function over 3 periods. For each of *n *genes, one of the *k *classes is randomly chosen, and using the expression profile *α *for the chosen class, the expression value *g*_*ij *_for gene *i *in experiment *j *is determined as *g*_*ij *_= *β*_*i*_*α*_*j *_+ *γ*_*i *_+ *ε*_*ij *_where *β*_*i *_and *γ *_*i *_are chosen uniformly at random. The error *ε*_*ij *_is randomly selected from *N*(0, σji2
 MathType@MTEF@5@5@+=feaafiart1ev1aaatCvAUfKttLearuWrP9MDH5MBPbIqV92AaeXatLxBI9gBaebbnrfifHhDYfgasaacH8akY=wiFfYdH8Gipec8Eeeu0xXdbba9frFj0=OqFfea0dXdd9vqai=hGuQ8kuc9pgc9s8qqaq=dirpe0xb9q8qiLsFr0=vr0=vr0dc8meaabaqaciaacaGaaeqabaqabeGadaaakeaaiiGacqWFdpWCdaqhaaWcbaGaemOAaOMaemyAaKgabaGaeGOmaidaaaaa@324D@). The variances σji2
 MathType@MTEF@5@5@+=feaafiart1ev1aaatCvAUfKttLearuWrP9MDH5MBPbIqV92AaeXatLxBI9gBaebbnrfifHhDYfgasaacH8akY=wiFfYdH8Gipec8Eeeu0xXdbba9frFj0=OqFfea0dXdd9vqai=hGuQ8kuc9pgc9s8qqaq=dirpe0xb9q8qiLsFr0=vr0=vr0dc8meaabaqaciaacaGaaeqabaqabeGadaaakeaaiiGacqWFdpWCdaqhaaWcbaGaemOAaOMaemyAaKgabaGaeGOmaidaaaaa@324D@ for each component *j *of *g_i _*are sampled from the inverse gamma distribution which approximates the error components of the yeast expression data set [57].

### Real expression data

Two sets of real expression data are employed, one from Affymetrix oligonucleotide microarrays assaying expression of *E. coli *genes and the second from cDNA microarrays assaying expression of yeast genes. The first set of expression data consists of results from 55 experiments under a battery of different conditions [65-68]. Typically, each *E. coli *gene is assayed with 15 probes. A detailed description of the array design has been described elsewhere [65], and the raw data is available from a public repository [69]. The expression values and corresponding standard errors are calculated using the expectation-maximization approach of Li and Wong [31] and are available as supplemental material. This data set is appropriate for two reasons. First, the data contains repeat measurements for each gene, which provides standard error estimates. As researchers are placing increasing emphasis on designing reproducible array experiments with suitable error models, replicate measurements are becoming more common. Second, we develop an external evaluation criterion for this data set which allows validation of the clustering results. Developing evaluation metrics for clustering real expression data is often challenging because there is rarely a *gold standard *indicating which genes should cluster together and which genes should be in different clusters. In order to validate the approach on the *E. coli *data, RegulonDB [55] and EcoCyc [56] were queried to identify 904 genes which have identifiable (non-constant) expression profiles and which have been experimentally verified as belonging to multi-gene operons. These genes belong to 275 operons and serve as a reasonable external standard under the assumption that genes expressed as part of the same polycistronic mRNA should be co-regulated and should belong to the same *class*.

The second set of data consists of expression measurements from 80 experiments corresponding to 20 perturbations of the galactose utilization pathway in *Saccharomyces cerevisiae*, assayed in quadruplicate [57]. Each of the yeast genes has been annotated as corresponding to one of four functional classifications in the gene ontology [58]. The functional classifications serve as the external standard of classification.
